# Human papillomavirus genome variants and head and neck cancers: a perspective

**DOI:** 10.1186/s13027-018-0185-6

**Published:** 2018-04-10

**Authors:** Jean-Damien Combes, Silvia Franceschi

**Affiliations:** 1 International Agency for Research on Cancer, 150 cours Albert Thomas, 69372 Cedex 08 Lyon, France; 2 Cancer Epidemiology Unit, CRO Aviano National Cancer Institute IRCCS, Via Franco Gallini 2, 33081 Aviano, PN Italy

**Keywords:** Human papillomavirus, HPV variants, HPV genome, Head and neck cancer, Epidemiology

## Abstract

Human papillomaviruses (HPV) cause infections that are responsible for diverse clinical manifestations from benign conditions to invasive cancer. As different HPV types are associated with variable pathogenic potential, minor genetic variations within a given high-risk HPV type might also be associated with distinct oncogenic capacities, through variable ability of persistence or risk of progression to precancer/cancer. Most recent HPV variant studies in the cervix using latest sequencing technology confirmed that minor changes in the HPV genome can have a major influence on carcinogenesis and have revealed key data that help better understand the carcinogenicity of HPV at a molecular level. Here we review the limited number of studies on HPV genome variants in head and neck cancers (HNC) and discuss their implications for cancer research in the light of accumulated knowledge for the cervix. Challenges in transposing HPV variant studies from the lower anogenital to the upper aerodigestive tract are also discussed, highlighting the main gaps of knowledge in the field of HPV-induced HNC. Specifically in the head and neck region, the lack of characterisation of precancerous lesions and the difficulty in sampling normal tissue will challenge the development of accurate studies. Although there is so far no indication that HPV variant research in HNC could directly translate into clinical application, such research is expected to be useful to disentangle unanswered questions in the pathogenesis of HNC. Yet, history of HPV variant research suggests that, to be successful, studies will require large international collaborative efforts.

## Background

Human papillomaviruses (HPV) cause infections that are responsible for diverse clinical manifestations from warts (papillomas) to invasive cancer. A dozen high-risk (HR) HPV types are powerful human carcinogens and the primary cause of cancer of the cervix and anogenital tract [1]. In the upper aerodigestive tract, HPV16 is recognized as the cause of a growing proportion of cancer of the oropharynx, particularly in the tonsil and the base of the tongue, although with substantial international variations [2].

It is well established that although all HPV are genetically related, their pathogenic characteristics differ widely [1]. As different HR-HPV types are associated with variable pathogenic potential [3], minor genetic variations within a given HR-HPV type might also be associated with distinct oncogenic capacities, through variable ability of persistence or risk of progression to precancer/cancer [4]. With recent improvement in DNA sequencing technology [5], promising findings were reported on the influence of HPV variants in carcinogenesis in cervical cancer that has been much more extensively studied than head and neck cancer (HNC) [6, 7], opening potential scope for clinical applications.

In the present article, we reviewed the limited number of studies on HPV genome variants in HNC and discussed their implications for cancer research in the light of accumulated knowledge in the cervix. Challenges in transposing HPV variant studies from cervical to HNC are also discussed, highlighting the main gaps of knowledge in the field of HPV-induced HNC.

### Terminology

Papillomaviruses are small non-enveloped viruses with circular double-stranded DNA of around 7000–8000 nucleotides infecting skin and mucosa of a variety of mammals, reptiles and birds [1]. Papillomaviruses are highly species-specific and are considered to have coevolved with their host since their origin, for hundreds of millions of years. The stability of the double-stranded structure of the genome results in a low mutation rate and it is considered that it takes millions of years for sequential accumulation of genetic changes to become fixed, leading to distinct HPV types [8].

Papillomaviruses are subdivided in genus, species, types and subtypes according to degree of viral genetic variation. Evaluation of differences in the L1 open reading frame DNA sequence is considered sufficient up to type-level classification, as it is accepted that L1 is robust enough to fully determine these subdivisions [9]. Differences of more than 40% between 2 HPV sequences define different “genus” (e.g., *Alphapapillomavirus*, *Betapapillomavrius*), differences of 30–40% define “species” (e.g., *Alphapapillomavirus* 7, *Alphapapillomavirus* 8), and differences of 10–30% define “types” (e.g., human papillomavirus 16 belonging to *Alphapapillomavirus* 9). Of note, the International Committee on Taxonomy of Viruses provides a taxonomic nomenclature only up to the species level [10].

HPV “variants” are smaller genetic variations in the viral DNA sequence within a given HPV type. At the subtype level, the evaluation of the difference in the whole genome sequence is considered necessary [9]. Differences between 1 and 10% define “lineages” (e.g., HPV16_A, HPV16_B) and differences between 0.5 to 1% define “sublineages” (e.g., HPV16_A1, HPV16_A2). The terminology is, however, evolving rapidly following progress in molecular biology and often hampers appropriate comparisons across studies. Previously used HPV variant classification referring to geography (e.g., “African-1”, “Asia-American”, “European”) corresponds to population groups where each lineage is most often found, but, although being practical, the use of this terminology is no longer recommended [9, 11]. Sublineages A1, A2 and A3 correspond to previously termed “European” lineage; lineages B and C to “African-1” and “African-2”, respectively, sublineage D1 to “North-American”, sublineages D2 and D3 to “Asian-Amercian”, and sublineage A4 to “Asian”.

At the subtype level, minor genetic variations that do not fit a phylogenetic tree are also characterised (hereinafter referred to as “non-lineage-specific HPV variants”) and correspond to more recent mutations. These non-synonymous single nucleotide changes that can appear independently from lineages are characterised by their DNA or amino acid substitution (e.g., HPV16 T350G located on E6 gene corresponds to L83 V amino acid change).

### Early studies on HPV variants and cervical cancer

All twelve HR-HPV belong to the *Alphapapillomavirus* genus (species 5, 6, 7 or 9) but widely differ in prevalence (related to evolutionary fitness) and risk of causing precancer/cancer. In the same manner, intra-type genetic variations might present differential pathogenic properties, through variable capacity to trigger immune response, ability to persist, or risk of progression to precancer/cancer. It is, for instance, conceivable that a minor variation in the E6 or E7 sequence may induce differential propensity of the corresponding protein to bind p53 or pRB and impact the risk of progression to cancer by modifying their capacity to inactivate the corresponding tumour suppressor functions.

Risk associated to HPV variants in cervical carcinogenesis has been studied since the early 1990s [12, 13]. There is a substantial accumulation of data from epidemiologic and mechanistic studies on the influence of various HPV variants in cervical pathogenesis. Historically, HPV variants in the cervix were compared for “European” versus “non-European” HPV lineages (“A” vs. “B/C/D” (sub)lineages following the most recent nomenclature). The “non-European” HPV16 lineages have been generally found to be associated with higher persistence [14, 15] and higher progression to cancer [14–20] compared to “European” lineages, most often in studies from Europe and USA. Another well-studied HPV variant, T350G, is non-lineage-specific and corresponds to a single nucleotide change in the HPV16 E6 gene. HPV16 350G was similarly associated with higher persistence [21–23] and progression to cancer [24–28] compared to HPV16 350 T. Some experimental and mechanistic evidence has partly supported the plausibility of these associations [28–36]. Other studies have also suggested differential risk of glandular vs. squamous cancers associated with specific HPV lineages [20, 37, 38].

However, globally, early studies on HPV variants in the cervix were judged as relatively disappointing. Inadequate sample size probably partly explains the inconsistencies between these studies with regard to the direction of the variants’ effect, but also prevented further evaluation of these observations [39–47]. Indeed, functional differences might be attributed not only to the effect of one isolated genetic variation but to specific combinations of amino acid changes. In fact, early studies had already by that time strongly suggested that the observed increased pathogenicity related to some HPV variants could be specific to a population [48–50] because of host-related factors [42, 51–53].

### Next-generation sequencing era and studies on HPV variants

With recent development of next-generation methods, their increasing availability and adaptability to large-scale populations, promising findings have emerged on pathogenic effects related to HPV variants in cervical cancer. These greatly improved approaches pave the way for the evaluation of variants in other HPV-associated cancer sites such as cancer of the oropharynx.

Mirabello et al. evaluated the association between HPV16 lineages and risk of precancer/cancer in 3200 women from a US cohort [6], using a whole genome sequencing assay optimized for HPV genome sequencing [5]. This study confirmed the early observation of a higher risk of precancer/cancer associated with B/C/D as a group compared to A lineages. Most importantly, further stratification by sublineage and by specific histologic outcome was possible due to appropriate sample size. In this case-control analysis (controls being HPV16-positive women without cervical intraepithelial neoplasia (CIN) grade 2+ after ≈3 years follow-up), it was shown that the overall association between HPV16 lineage and cervical cancer risk masked strong heterogeneity in pathogenicity according to sub-lineage and disease outcome.

Indeed, it was shown that previously defined “European” variants actually regrouped sublineages with substantially different risks of precancer/cancer. For instance, risk associated with sublineage A4 was markedly higher compared to A1/A2. In the same manner, risk associated with histology outcome showed strong heterogeneity. Odds ratio (OR) of glandular cancer for D2 vs A1/A2 sublineages was 137.3 (95% CI: 37.2–506.9) whereas OR of squamous cancer was 7.6 (95%CI: 1.4–39.8). This finding was corroborated by a comparable study using samples collected worldwide [54]. Although the absolute risk of cervical adenocarcinoma remains low, such a high effect size points to possibilities for a clinical application, given the difficulty to identify glandular lesions by cytology and a poorer prognosis compared to squamous type.

In addition, this study confirmed the early observation that some variants present a higher carcinogenic effect in women whose genetic background corresponds to that of the virus. For instance, Caucasian white women infected with A1/A2 variant were at a higher risk of CIN3+ compared to women of other genetic backgrounds. Similarly, Asian and Hispanic women had increased risk, although non-significant, associated with A4 and D2/D3 sublineages compared with other races/ethnicities. Of note, the magnitude of the effect of associations with genetic backgrounds was relatively low (OR≈1.5).

An important and unexpected finding came from the same collaborative group who analysed more than 5000 HPV16 case control samples worldwide [7]. It was shown that the HPV16 E7 sequence (98 amino acids) leading to cervical cancer is virtually invariant compared to high sequence variability in controls. This finding was confirmed to be consistent across regions and ethnicity. Of note, an earlier study has also suggested that the E7 sequence of HPV type 16 was less variable compared to other high risk types (HPV31) [55]. Although to be confirmed, a strict conservation of E7 could represent a promising highly specific biomarker and may also be important for HPV-associated and for non-cervical cancers.

### HPV variant studies and head and neck cancers

HNC includes numerous tumours that generally share strong associations with tobacco and alcohol consumption [56]. HPV16 is generally accepted as a carcinogen in tonsil and base of the tongue, but its implication in other sites such as oral cavity, larynx or even in oropharyngeal tissues outside the Waldeyer’s ring is at most a weak one [57, 58]. It is nonetheless conceivable that many non-tonsillar HNC are falsely classified as HPV-positive or are actually misclassified tonsillar or oropharyngeal cancers (OPC), as characterisation of the true site of origin is often difficult due to fast local extension and unclear anatomical boundaries.

HPV-induced OPCs involve both genders, although with higher incidence in men compared to women [59]. This sex-ratio is mainly explained by the higher HPV transmission for vaginal-oral rather than penile-oral sexual intercourse. Saunders et al. recently showed that risk of OPC was higher in women having sex with women compared with heterosexual women, although this association was not found in men having sex with men, in agreement with a higher risk of HPV transmission by vaginal- vs. penile-oral sex [60]. The lower risk of HPV-induced OPC observed in women could also be partly explained by the higher immunity acquired by women due to more frequent exposure to HPV in the genital mucosa and by a still little understood role of the combined exposure to HPV and tobacco that is generally stronger in men than women [59]. As for cervical cancer, the presence of other risk factors and host-characteristics should be considered in HPV variant studies of HNC.

Few studies reporting HPV16 variants in HNC are available (Table 1), including on the distribution of HPV variant lineages [61–67], T350G [61, 66, 68, 69] and other non-lineage-specific variants [61, 66, 67, 70]. These studies resemble early studies of cervical cancer in being mere descriptions of variant prevalence in small populations from North America and Europe. Some of these studies did not present data separately for oropharyngeal and other head and neck sites, and the definition of HPV-induced HNC is variable, using frequently only HPV-DNA detection or p16-positivity. These major limitations prevented us from any interpretation, thus our report regarding those studies remains descriptive.

**Table 1:** Studies on HPV16 variants in head and neck cancers

Study	Country	N HPV16 + samples	Seq.	Variant lineage (n)	Non lineage specific variants (n)
Gillison 2000 [67]	USA	52 HNC	E6	39 Eur; 9 Asian; 2 NA; 1 Afr-1	20 T350G 6 A131G
Hoffmann 2004 [70]	DE	21 HNC (5 OPC)	E6/E7		8 T350G (2 in OPC) 7 A131G (1 in OPC)
Badaracco 2007 [62]	IT	13 HNC (5 TC)	L1	9 Eur 2 Af-2; 1 AA	
Agrawal 2008 [61]	USA	14 HNC	E6	13 Eur 1 As	3 T350G
Boscolo Rizzo 2009 [68]	IT	8 HNC (4 OPC)	E6		5 T350G
Blakaj 2012 [65]	USA	43 HNC (28 OPC)	E6	31 Eur 7 AA; 3 Af	
Du 2012 [66]	SW	108 TC	E6	51(/55) EUR	43 T350G 21 A131G
Barbieri 2014 [63]	IT	51 OPC	L1	41 Eur 10 Af	
Hassani 2015 [69]	JP	10 TC	E6		8 T350G (1/3 T350G in tonsillitis)
Betiol 2016 [64]	BR	21 HNC (3 OPC)	E6	12 Eur 9 AA or NA1	

Abbreviations: *HNC* Head and neck cancer, *OPC* Oropharyngeal cancer

Countries: *BR*Brazil, *DE* Germany, *IT* Italia, *JP*, Japan, *SW* Sweden

HPV variant lineages: *EUR* European, *A* Asian, *AA* Asian American, *NA* North American, *Af* African

In an early study on the role of HPV in HNC, Gillison et al. provided data on prevalence of HPV variant lineages, T350G and other non-lineage-specific variants among 52 HPV16-positive HNC from the USA [67]. The observed distribution of HPV variants was judged similar to that in a contemporary study of cervical cancers in North America. In a comprehensive study on oral HPV infection before and after treatment, Agrawal et al. reported HPV variant lineage distribution as well as T350G and T131G in patients diagnosed with HPV-induced HNC in the USA. In the latter study, oral rinses were also collected and E6 sequence identity was compared with the tumour (concordant in 10/11) [61].

Blakaj et al. have evaluated the association between variant lineages and HNC disease stage, hypothesising differential variant distribution in higher TNM and N+ staged tumours [65]. Barbieri et al. have also compared clinical stage according to HPV variant lineages in 51 OPC cases from Italy but failed to detect any association [63]. Unexpectedly in this study, African lineage was detected in 10 out of 51 OPC. In Hassani et al., frequency of T350G was reported in 10 HPV16-positive tonsillar cancer and 3 HPV-positive tonsillitis specimens [69]. The same team had also previously compared distribution of HPV variant lineages and non-lineage-specific variants in Japan, Pakistan and Columbia in oral cavity and oesophageal cancer, but not in OPC [71].

One notable study compared E6 variants A131G (R10G) and T350G in 108 tonsillar and cervical cancers in Swedish patients [66]. In this study, a significantly higher representation of A131G was reported in tonsillar cancer (21/108) compared to cervical samples (2/51) and cervical cancer (0/52). The role of A131G is not clearly established but has been linked to p53 binding and degradation [72]. Of note, among other findings, presence of A131G variant was not associated with disease-free survival and T350G variant was common in tonsillar cancer (45%), cervical cancer (31%) and cervical samples (29%).

### Challenges and perspectives in studying HPV variants in head and neck cancers

Critical differences between genital and upper aerodigestive tracts need to be underlined [73, 74], implying specific challenges in research on HPV variants. Cervical cancer is nearly always caused by HPV and, worldwide, it is a much more frequent cancer than HPV-induced HNC [2]. Also importantly, the collection of cervical samples at different steps of carcinogenesis is relatively easy for anatomical reasons and the long going practice of screening around the world. In the cervix, it is thus possible not only to analyse cancer cases but also longitudinal data at the individual level to evaluate the risk of persistent infection or progression to precancer/cancer associated with specific HPV variants.

The major challenge for the head and neck consists in the lack of characterisation of the carcinogenetic steps from normal tissue to cancer. Although few studies have attempted to collect precancerous lesions in non-cancerous patients using cytology from in vivo [75–77] or ex vivo [78] tonsillar brushings, none were successful. There is however a clue that a precancerous state exists, and long before the diagnosis of cancer. Two longitudinal studies evaluating HPV16 serology reported not only a high specificity but most importantly that in OPC cases, HPV16 seropositivity could be detected more than 10 years prior to diagnosis [79, 80]. Yet the suspected precancerous lesions are hardly identifiable most probably because HPV-induced tonsillar cancer is believed to arise from the depth of the crypts and is hence challenging to sample [78].

An additional critical challenge exists when trying to assess whether certain HPV variants show differential risk of persistent infection. Indeed sampling non-cancerous tonsillar or oropharyngeal tissue to detect HPV infection is problematic. All sampling methods are imperfect due to specific limitations [81, 82]: evaluation of frozen or paraffin biopsies suffers from a lack of exhaustiveness; although more representative than a biopsy; oropharyngeal brushing hardly permits sampling inside the crypt; rinse/gargle does not inform on what tissue is evaluated and gargle can be impossible to some patients due to laryngeal spasm in addition to the uncertainty that even a proper gargle can detect an infection inside a crypt. We recently showed that concordance between HPV detection using rinse/gargle, tonsil ex vivo brushing and frozen biopsies is critically low [81, 82]. Accordingly, studies of the natural history of HPV in the oropharynx have not been possible so far.

Yet case-case comparisons including other HPV-induced cancer sites should be informative [11]. The greater predominance of HPV16 in OPC (around 90%) compared to the cervix (50/60%) suggests a different host-viral interaction in the two sites. It is therefore credible that some HPV variants without influence in cervical pathogenesis could play a role in the oropharynx, as specific sublineages are associated with specific histological subtypes [6], some HPV variants could be more prone to infect or to trigger progression to cancer in oropharyngeal compared to cervical tissue. Likewise, although there is no data so far suggesting that HPV variants could have an effect on therapeutic response, an influence is possible, for instance through modification of the tumour microenvironment.

There is so far no indication that HPV variant research in HNC would be directly clinically relevant. However, such research could be useful to disentangle other unanswered questions including HPV genome integration [83–85], the identification of a robust method to determine a truly HPV-driven HNC or those with the best prognosis [58, 86, 87], or a possible distinct pathogenesis in an immunosuppressed population. Of note, the use of complete genome sequencing obviously allows finer definition of persistent vs. cleared infection in longitudinal studies [88] or finer confirmation of the concordance of HPV detection in rinse/gargles with HPV-HNC tissue [61]. Other illustrations of HPV variant studies included evaluation of their influence on HPV serological response [89] or on HPV vaccine efficacy [90]. Regarding non-cancerous HPV-related conditions, such as recurrent papillomatosis or genital warts, yet unanswered questions might also take advantage of HPV variant studies [91–93].

## Conclusion

In conclusion, our review suggests that the most recent HPV variant studies in cervical cancer are of importance in planning the evaluation of HPV heterogeneity in other HPV-associated cancer including HNC. HPV variant studies using recent sequencing technology have generated key data that should help better understand the carcinogenicity of HPV at a molecular level. If these successful studies have confirmed that minor changes in the HPV genome can have a major influence on carcinogenesis, they also highlight the crucial need for international collaborative efforts to allow appropriate in depth analyses.

## Abbreviations

CI: confidence interval
CIN: Cervical intraepithelial neoplasia
HNC: head and neck cancer
HR-HPV: high-risk human papillomavirus virus
OPC: oropharyngeal cancers
